# The role of sex and rurality in cancer fatalistic beliefs and cancer screening utilization in Florida

**DOI:** 10.1002/cam4.4122

**Published:** 2021-07-13

**Authors:** Yi Guo, Sarah M. Szurek, Jiang Bian, Dejana Braithwaite, Jonathan D. Licht, Elizabeth A. Shenkman

**Affiliations:** ^1^ Department of Health Outcomes and Biomedical Informatics College of Medicine University of Florida Gainesville FL USA; ^2^ University of Florida Health Cancer Center Gainesville FL USA; ^3^ Department of Aging and Geriatric Research College of Medicine University of Florida Gainesville FL USA; ^4^ Department of Epidemiology College of Public Health and Health Professions and College of Medicine University of Florida Gainesville FL USA; ^5^ Division of Hematology and Oncology Department of Medicine College of Medicine University of Florida Gainesville FL USA

**Keywords:** colonoscopy, disparity, fecal occult blood test, mammography, sigmoidoscopy

## Abstract

**Background:**

People's fatalistic beliefs about cancer can influence their cancer prevention behaviors. We examined the association between fatalistic beliefs and breast and colorectal cancer screening among residents of north‐central Florida and tested whether there exists any sex or rural–non‐rural disparities in the association.

**Methods:**

We conducted a cross‐sectional, random digit dialing telephone survey of 895 adults residing in north‐central Florida in 2017. Using weighted logistic models, we examined the association between (1) respondents’ sociodemographic characteristics and cancer fatalistic beliefs and (2) cancer fatalistic beliefs and cancer screening utilization among screening eligible populations. We tested a series of sex and rurality by fatalistic belief interactions.

**Results:**

Controlling for sociodemographics, we found the agreement with “It seems like everything causes cancer” was associated with a higher likelihood of having a mammogram (odds ratio [OR]: 3.34; 95% confidence interval [CI]: 1.17–9.51), while the agreement with “Cancer is most often caused by a person's behavior or lifestyle” was associated with a higher likelihood of having a blood stool test (OR: 1.85; 95% CI: 1.12–3.05) or a sigmoidoscopy or colonoscopy among women (OR: 2.65; 95% CI: 1.09–6.44). We did not observe any rural–non‐rural disparity in the association between fatalistic beliefs and cancer screening utilization.

**Conclusions:**

Some, but not all, cancer fatalistic beliefs are associated with getting breast and colorectal cancer screening in north‐central Florida. Our study highlights the need for more research to better understand the social and cultural factors associated with cancer screening utilization.

## Graphical abstract

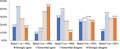

## 1 INTRODUCTION

As the second leading cause of death in the United States (US), cancer is responsible for one in almost every four deaths.^1^ It has been projected that about 1.9 million new cancer cases and over 600 thousand cancer‐related deaths will occur in the US in 2021.^2^ To reduce cancer mortality and alleviate the burden of cancer in the population, it is crucial to detect cancer in the early stages through cancer screening, which substantially increases the chance of successful treatment and provides the best opportunity for survival. The effectiveness of cancer screening in reducing cancer mortality has been proven in many clinical studies, including many randomized clinical trials.^3^, ^4^ Furthermore, cancer screening programs are proven to be cost‐effective and sometimes cost‐saving in economic evaluations.^5^, ^6^, ^7^, ^8^, ^9^ Given the extensive scientific evidence on the effectiveness of cancer screening, professional associations, such as the American Cancer Society (ACS) and the U.S. Preventive Services Task Force, have published guidelines that recommend cancer screening for at‐risk populations. These guidelines identify the populations who are most likely to benefit from screening and provide recommendations on the type and frequency of screening tests.

Moreover, people's fatalistic beliefs about cancer can greatly influence their cancer prevention behaviors such as getting screened for cancer.^10^, ^11^ Cancer fatalistic beliefs represent the negative perception that cancer development is beyond human control and death is inevitable if diagnosed with cancer.^10^ More specifically, fatalistic beliefs about cancer can include pessimism (e.g., "it seems like everything causes cancer"), helplessness (e.g., "there's not much you can do to lower your chances of getting cancer"), and confusion (e.g., “There are so many different recommendations about preventing cancer, it's hard to know which ones to follow”).^12^ Individuals who hold these fatalistic beliefs are more likely to avoid cancer risk information and less likely to engage in cancer prevention behaviors due to the perception of lack of control,^12^, ^13^ reduced self‐efficacy and motivation,^12^, ^14^ or them putting lower value on cancer prevention behaviors.^13^, ^15^

Furthermore, prior research has suggested that there exists sex and rural–non‐rural disparities in cancer fatalistic beliefs in the US, although the evidence on sex disparity is inconsistent and sometimes contradictory across studies. For example, multiple studies have reported a sex disparity in cancer fatalistic beliefs.^13^, ^16^, ^17^, ^18^, ^19^, ^20^ However, women and men do not consistently agree on a set of fatalistic belief statements, and the sex disparity in fatalistic beliefs seems to vary by geographic locations.^13^, ^16^, ^17^, ^18^, ^19^, ^20^ Regarding rural–non‐rural differences in cancer fatalistic beliefs, prior studies using the national representative or state‐level data have reported that rural residents are more likely to endorse fatalistic belief statements about cancer (e.g., “It seems like everything causes cancer”) than urban residents.^16^, ^21^

In this study, we aimed to examine the influence of sociodemographic factors on cancer fatalistic beliefs, and test whether the fatalistic beliefs are associated with undergoing breast and colorectal cancer screening utilization among individuals residing in a large southeastern academic medical university's catchment area in the north‐central Florida region. Considering the large geographic differences in sex disparity in cancer fatalistic beliefs and the highly rural nature of the north‐central Florida region, we also examined whether the impacts of fatalistic beliefs on cancer screening utilization varied by sex or rurality. Compared to the US and Florida, the catchment area has more people older than 65 years, more whites, fewer Hispanics, more rural areas, and a higher poverty rate, especially in the rural counties.^22^ In addition, the catchment area is disproportionally affected by cancer. Our calculations using the Florida Cancer Data System data have shown that the area has higher age‐adjusted cancer incidence, advanced cancer incidence, and cancer mortality than the US and Florida.^23^, ^24^ Considering the great evidence on sociodemographic disparities in cancer fatalistic beliefs, it is important to examine how fatalistic beliefs impact cancer screening utilization in this area of unique social and cultural characteristics as well as high cancer burden.

## 2 MATERIALS AND METHODS

### 2.1 Study design and survey methodology

**FIGURE 1:**
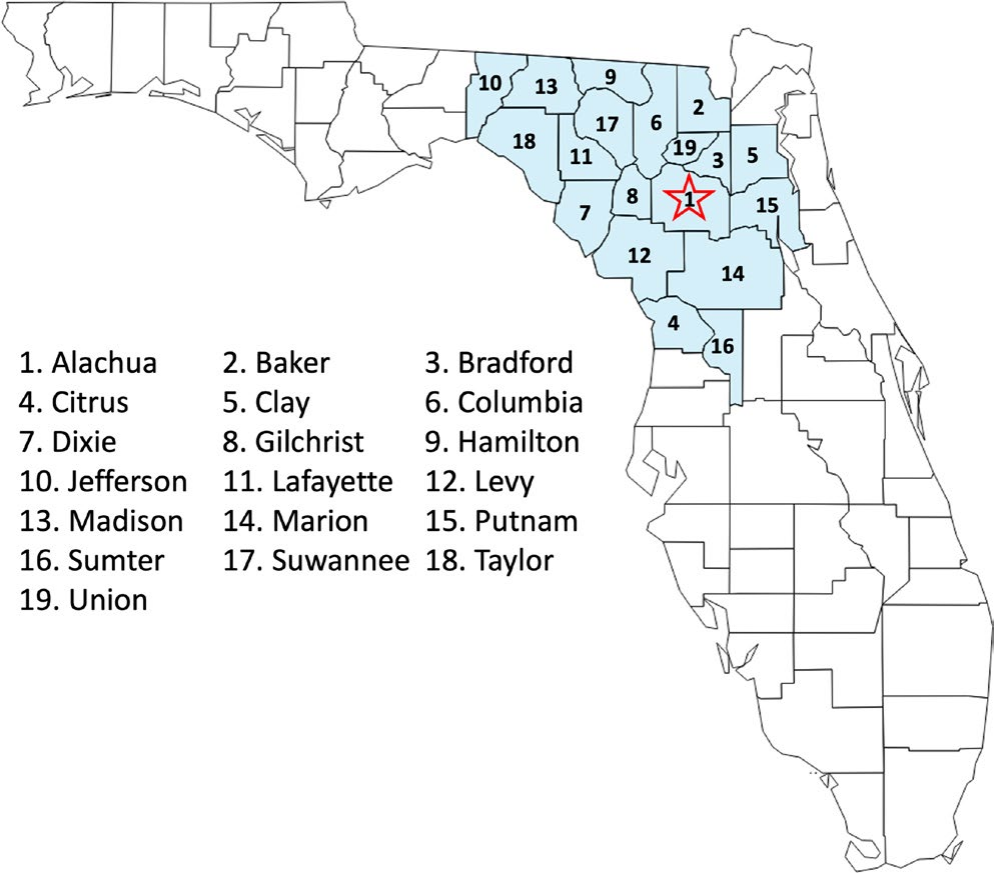
Counties in the study area in north‐central Florida. The 19 counties in the catchment area of the large southeastern academic medical university

Our study was a cross‐sectional, random digit dialing (RDD) telephone survey of 895 adults residing in 19 counties in the catchment area of a large southeastern academic medical university (Figure 1). We adapted items from the Behavioral Risk Factor Surveillance System and Health Information National Trends Survey (HINTS) in our survey to collect information on sociodemographics, cancer fatalistic beliefs, and cancer screening utilization.

We contracted with a professional survey center to conduct the RDD, which happened between June and September 2017. The survey center sampled from a list of phone numbers (80% mobile phones and 20% landlines) of individuals residing in the 19 counties purchased from a sampling firm. Each phone number was dialed a maximum of 15 (landline) or 6 (mobile phone) times, with at least 1 attempt during a weekday, weeknight, and weekend. To maximize the participation of Black individuals, we oversampled Blacks on a 3:1 ratio. A final survey weight was created as the product of the design, nonresponse, and calibration weights, and used in all data analysis. All surveys were administered by professional interviewers via the computer‐assisted telephone interviewing technique. The interviewers obtained verbal informed consent from all participants before completing the telephone surveys.

### 2.2 Variables of interest

#### 2.2.1 Cancer fatalistic beliefs

We assessed the participants’ agreement with five cancer belief statements adapted from the HINTS survey. The participants were asked “How much do you agree or disagree with each of the following statements?” and provided with five belief statements: (1) “It seems like everything causes cancer,” (2) “There's not much you can do to lower your chances of getting cancer,” (3) “There are so many different recommendations about preventing cancer, it's hard to know which ones to follow,” (4) “When I think about cancer, I automatically think about death,” and (5) “Cancer is most often caused by a person's behavior or lifestyle.” The response options were Strongly agree, Somewhat agree, Somewhat disagree, Strongly disagree, Don't know, or Refused. For each statement, we created a dichotomous variable to indicate whether the participants agreed (Strongly agree or Somewhat agree) or disagreed (Somewhat disagree or Strongly disagree) with the statement.

#### 2.2.2 Cancer screening utilization

We assessed the participants’ utilization of breast cancer screening (mammogram) and colorectal cancer screening (blood stool test or sigmoidoscopy/colonoscopy). Breast cancer screening was assessed in female participants with the question “A mammogram is an x‐ray of each breast to look for breast cancer. Have you ever had a mammogram?”. Colorectal cancer screening was assessed in all participants with two questions: “A blood stool test is a test that may use a special kit at home to determine whether the stool contains blood. Have you ever had this test using a home kit?” and “Sigmoidoscopy and colonoscopy are exams in which a tube is inserted in the rectum to view the colon for signs of cancer or other health problems. Have you ever had either of these exams?”. The response options were Yes, No, Don't know/Not sure, or Refused for these questions. For each screening test, we created a dichotomous variable to indicate whether the participants had ever had the test (Yes or No).

#### 2.2.3 Sociodemographic characteristics

We included the following sociodemographic variables in this study: age, sex (Women or Men), race (White, Black, or Other), Hispanic origin (Yes or No), education (High school or lower or More than high school), household income (lower than $35,000, $35,000 to under $75,000, or $75,000 or more), rural residency (Rural or Non‐rural), current cigarette smoking (Yes or No), and health insurance coverage (Covered or Uncovered). Household income was assessed with the question “Thinking about members of your family living in this household, what is your combined annual income, meaning the total pre‐tax income from all sources earned in the past year?” and categorized into lower than $35,000, $35,000 to under $75,000, or $75,000 or more. We determined whether a participant was a rural or non‐rural resident based on the county information collected in the survey and the state of Florida’s definition of “rural county” according to the Florida Statutes Section 288.0656.^25^ Current cigarette smoking (Yes or No) was assessed with two questions adapted from HINTS and operationalized as having ever smoked 100 cigarettes and currently smoking cigarettes every day or some days.

### 2.3 Statistical analysis

Statistical analysis was conducted in three parts. First, we calculated the frequencies and survey‐sampling weighted percentages of the variables of interest to describe our study sample. Second, we examined the association between respondents’ characteristics and cancer fatalistic beliefs in separate weighted multivariable logistic regression models. In each model, the dependent variable was in agreement with the belief statement (agree vs. disagree) and the independent variables were the sociodemographic variables: age, sex, race, Hispanic origin, education, household income, rural residency, current cigarette smoking, and health insurance coverage. Third, we built weighted multivariable logistic regression models to examine the association between cancer fatalistic beliefs and cancer screening utilization among screening eligible populations. Based on the ACS cancer screening guidelines,^26^, ^27^ we defined the population eligible for breast cancer screening as female respondents aged 40 or older, and the population eligible for colorectal cancer screening as respondents aged 45 years or older but younger than 76. For each of the screening tests (mammogram, blood stool test, or sigmoidoscopy/colonoscopy), we built a weighted multivariable logistic regression model with the independent variables being the sociodemographic variables and the agreement with each of the belief statements. To examine if there existed any sex or rural–non‐rural disparities in the association between cancer fatalistic beliefs and cancer screening utilization, we included and tested a series of sex or rural residency by agreement with cancer fatalistic belief interactions in all logistic models. Non‐significant interactions (*p* > 0.05) were excluded from the models. Results from all logistic models were reported as odds ratios (ORs) and the associated 95% confidence intervals (CIs). All analyses were conducted using SAS version 9.4 (SAS Institute, Inc., Cary, NC, USA).

## 3 RESULTS

### 3.1 Respondents’ characteristics

We summarized the frequencies and weighted percentages of the sociodemographic variables in Table 1. Over 90% of the respondents were aged 75 years or younger; 45.6% were younger than 50, and 44.6% were between the age of 50 and 75. There was a slightly higher percentage of female (52.8%) than male (47.2%) respondents in our study sample. The majority of the respondents were white (76.4%), non‐Hispanic (94.4%), had more than high school education (62.0%), resided in non‐rural counties (77.9%), did not currently smoke (78.9%), and had health insurance coverage (79.9%). Regarding annual household income, 39.7% of the respondents reported an income lower than $35,000, 32.4% reported an income between $35,000 and $75,000, and 27.9% reported an income of $75,000 or more. The demographic characteristics of the study sample matched well with those reported for the 19 counties in the catchment area by the Census Bureau. According to the U.S. Census 2017 American Community Survey 5‐year estimates,^22^ these 19 counties were 90.1% younger than 75 years, 50.3% female, 80.2% White, and 91.4% non‐Hispanic.

**TABLE 1:** Respondents’ characteristics

	*n*	Weighted %
Age		
18–49	338	45.6
50–75	469	44.6
>75	80	9.8
Sex		
Women	502	52.8
Men	393	47.2
Race		
White	604	76.4
Black	160	16.3
Other	119	7.3
Hispanic origin		
Yes	56	5.7
No	830	94.4
Education		
≤HS	237	38.0
>HS	647	62.0
Household income		
<$35k	289	39.7
$35k–$75k	263	32.4
≥$75k	253	27.9
Rural residency		
Rural	111	22.1
Non‐rural	784	77.9
Current smoking		
Yes	134	21.1
No	758	78.9
Health insurance		
Covered	756	79.9
Uncovered	132	20.1

Abbreviation: HS, high school.

**FIGURE 2:**
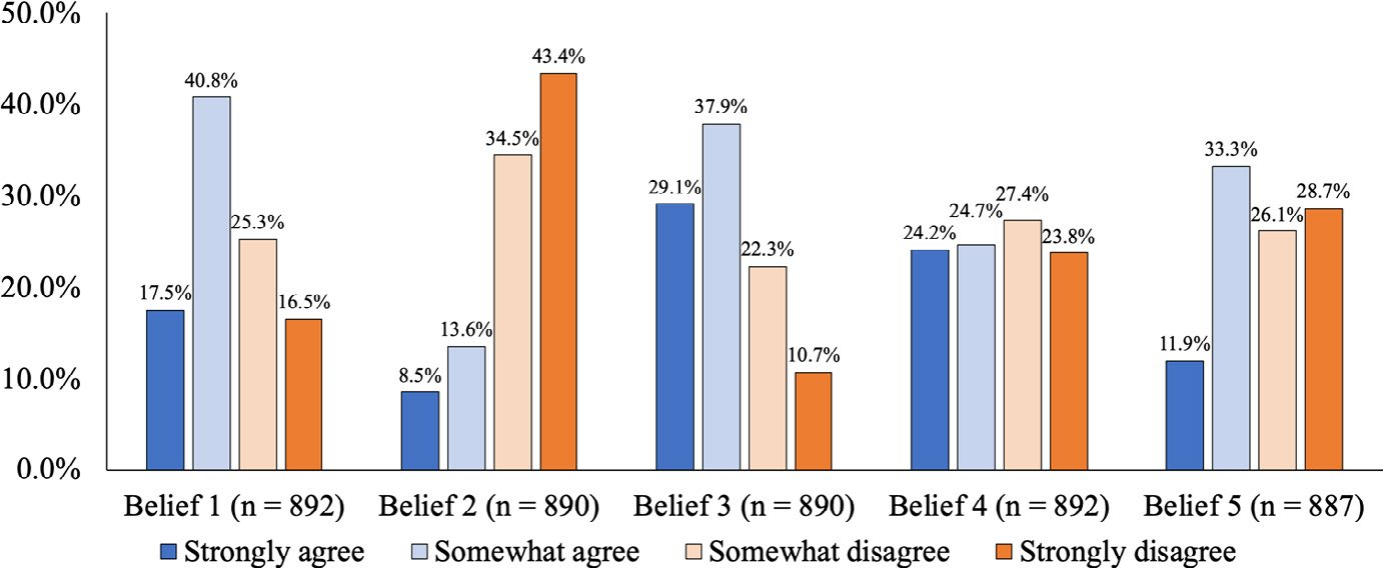
Weighted distributions of the cancer fatalistic beliefs. Number and weighted percentages of participants responding to each of the cancer fatalistic belief questions. Belief 1: It seems like everything causes cancer. Belief 2: There is not much you can do to lower your chances of getting cancer. Belief 3: There are so many different recommendations about preventing cancer, it is hard to know which ones to follow. Belief 4: When I think about cancer, I automatically think about death. Belief 5: Cancer is most often caused by a person's behavior or lifestyle

We summarized the number and weighted percentages of participants responding to each of the cancer fatalistic belief questions in Figure 2. Over half of the respondents (58.3%) agreed with the belief “It seems like everything causes cancer.” Most respondents disagreed (77.9%) with the belief “There's not much you can do to lower your chances of getting cancer,” but agreed (67.0%) with the belief “There are so many different recommendations about preventing cancer, it's hard to know which ones to follow.” A roughly equal percentage of respondents agreed (48.9%) and disagreed (51.1%) with the belief “When I think about cancer, I automatically think about death.” A slightly lower percentage of respondents agreed (45.2%) with the belief “Cancer is most often caused by a person's behavior or lifestyle.”

### 3.2 Association of respondents’ characteristics with cancer fatalistic beliefs

We summarized results from the weighted multivariable logistic regression on cancer fatalistic beliefs in Table 2. Adjusting for the other variables, older age was associated with a lower likelihood of agreeing with “It seems like everything causes cancer” (OR [age 50–75 vs. age 18–49] = 0.72; 95% CI: 0.52–0.99 and OR [age > 75 vs. age 18–49] = 0.38; 95% CI: 0.22–0.67) and “There are so many different recommendations about preventing cancer, it's hard to know which ones to follow” (OR [age > 75 vs. age 18–49] = 0.55; 95% CI: 0.32–0.97). Men were more likely than women to agree with “It seems like everything causes cancer” (OR = 1.39; 95% CI: 1.02–1.90) and “Cancer is most often caused by a person's behavior or lifestyle” (OR = 2.33; 95% CI: 1.72–3.15). Blacks were more likely than Whites to agree with “There's not much you can do to lower your chances of getting cancer” (OR = 1.95; 95% CI: 1.25–3.03). Hispanics were more likely than non‐Hispanics to agree with “There's not much you can do to lower your chances of getting cancer” (OR = 2.05; 95% CI: 1.01–4.17). However, we did not observe any other sex or racial–ethnic difference in the agreement with these belief statements.

**TABLE 2:** The association between respondents’ characteristics and cancer‐fatalistic beliefs in multivariable logistic models

	“It seems like everything causes cancer”	“There's not much you can do to lower your chances of getting cancer”	“There are so many different recommendations about preventing cancer, it's hard to know which ones to follow”	“When I think about cancer, I automatically think about death”	“Cancer is most often caused by a person's behavior or lifestyle”
	OR (95% CI)	OR (95% CI)	OR (95% CI)	OR (95% CI)	OR (95% CI)
Age					
50–75 versus 18–49	0.72 (0.52–0.99)	0.93 (0.64–1.36)	0.86 (0.62–1.19)	0.78 (0.58–1.06)	0.79 (0.58–1.08)
>75 versus 18–49	0.38 (0.22–0.67)	1.76 (0.95–3.25)	0.55 (0.32–0.97)	0.78 (0.45–1.35)	1.09 (0.62–1.90)
					
Sex					
Men versus Women	1.39 (1.02–1.90)	1.19 (0.83–1.71)	0.77 (0.57–1.05)	0.97 (0.72–1.30)	2.33 (1.72–3.15)
					
Race					
Black versus White	1.32 (0.86–2.02)	1.95 (1.25–3.03)	0.82 (0.54–1.25)	1.43 (0.96–2.13)	0.93 (0.61–1.41)
Other versus White	0.95 (0.51–1.79)	0.72 (0.33–1.59)	0.87 (0.47–1.61)	0.89 (0.49–1.61)	0.99 (0.54–1.83)
					
Hispanic origin					
Yes versus No	1.10 (0.55–2.20)	2.05 (1.01–4.17)	0.80 (0.41–1.56)	1.69 (0.86–3.31)	1.96 (0.99–3.90)
					
Education					
>HS versus ≤HS	0.66 (0.47–0.92)	0.74 (0.52–1.07)	1.05 (0.75–1.45)	0.95 (0.70–1.30)	0.78 (0.56–1.07)
					
Household income					
$35k–$75k versus <$35k	0.68 (0.48–0.99)	0.92 (0.61–1.38)	0.90 (0.62–1.31)	0.79 (0.56–1.12)	1.22 (0.85–1.75)
≥$75k versus < $35k	0.79 (0.53–1.18)	0.64 (0.39–1.02)	0.67 (0.46–0.99)	0.77 (0.53–1.12)	1.50 (1.02–2.21)
					
Rural residency					
Rural versus Non‐rural	1.11 (0.77–1.61)	1.22 (0.81–1.84)	1.10 (0.76–1.60)	1.29 (0.91–1.82)	1.47 (1.03–2.09)
					
Current smoking					
Yes versus No	1.99 (1.31–3.03)	1.60 (1.04–2.46)	1.41 (0.93–2.13)	1.29 (0.88–1.87)	1.15 (0.78–1.69)
					
Health insurance					
Covered versus Uncovered	0.63 (0.40–0.98)	0.98 (0.62–1.55)	1.42 (0.93–2.15)	0.91 (0.61–1.36)	0.88 (0.59–1.33)

Abbreviations: CI, confidence interval; HS, high school; OR, odds ratio.

Having more than high school education was associated with a lower likelihood of agreeing with “It seems like everything causes cancer” (OR = 0.66; 95% CI: 0.47–0.92), but did not significantly impact agreement with the other beliefs. Respondents with a household income between $35,000 to under $75,000 were less likely to agree with “It seems like everything causes cancer” (OR = 0.68; 95% CI: 0.48–0.99) compared to those with a household income lower than $35,000. Respondents with a household income of $75,000 or more were less likely to agree with “There are so many different recommendations about preventing cancer, it's hard to know which ones to follow” (OR = 0.67; 95% CI: 0.46–0.99) but more likely to agree with “Cancer is most often caused by a person's behavior or lifestyle” (OR = 1.47; 95% CI: 1.03–2.09) compared to those with a household income lower than $35,000. Rural residency was associated with a higher likelihood of agreeing with “Cancer is most often caused by a person's behavior or lifestyle” only (OR = 1.47; 95% CI: 1.03–2.09). Smokers were more likely to agree with “It seems like everything causes cancer” (OR = 1.99; 95% CI: 1.31–3.03) and “There's not much you can do to lower your chances of getting cancer” (OR = 1.60; 95% CI: 1.04–2.46) compared to non‐smokers. Last, health insurance coverage was associated with a lower likelihood of agreeing with “It seems like everything *causes cancer*” (OR = 0.63; 95% CI: 0.40–0.98).

### 3.3 Association of cancer‐fatalistic beliefs with cancer screening utilization

We summarized results from the weighted multivariable logistic regression on cancer screening utilization in Table 3. A total of 378 female respondents were eligible for breast cancer screening according to the ACS cancer screening guideline. Among these screening‐eligible women, 70.6% had ever had a mammogram. In the multivariable analysis, none of the sex or rural residency by agreement with cancer fatalistic belief interactions were significant. After dropping the interaction terms from the model, agreement with “It seems like everything causes cancer” was associated with a higher likelihood of having had a mammogram (OR = 3.34; 95% CI: 1.17–9.51) controlling for the other variables. Furthermore, older age was associated with a higher likelihood of having had a mammogram (OR = 1.09; 95% CI: 1.04–1.14). Having more than high school education was also associated with a higher likelihood of having had a mammogram (OR = 7.04; 95% CI: 2.45–20.2).

**TABLE 3:** The association between cancer fatalistic beliefs and cancer screening utilization in multivariable logistic models

	Ever had a mammogram (weight % = 70.6%)	Ever had a blood stool test (weight % = 38.0%)	Ever had a sigmoidoscopy or colonoscopy (weight % = 78.4%)
			
Age	1.09 (1.04–1.14)	1.08 (1.04–1.11)	1.08 (1.03–1.12)
			
Sex			
Men versus Women	NA	1.06 (0.65–1.72)	NA
			
Race			
Black versus White	0.51 (0.16–1.61)	1.43 (0.70–2.90)	2.09 (0.88–4.94)
Other versus White	0.96 (0.07–13.3)	2.01 (0.65–6.25)	0.62 (0.16–2.40)
			
Hispanic origin			
Yes versus No	0.85 (0.12–6.19)	0.97 (0.25–3.81)	6.13 (0.47–80.4)
			
Education			
>HS versus ≤HS	7.04 (2.45–20.2)	1.54 (0.90–2.64)	1.90 (1.06–3.41)
			
Household income			
$35k–$75k versus <$35k	1.79 (0.62–5.10)	0.83 (0.46–1.49)	1.89 (0.96–3.72)
≥$75k versus <$35k	2.48 (0.56–11.1)	0.77 (0.40–1.50)	2.28 (1.05–4.93)
			
Rural residency			
Rural versus Non‐rural	0.94 (0.27–3.30)	0.65 (0.36–1.15)	1.05 (0.55–2.00)
			
Current smoking			
Yes versus No	0.66 (0.23–1.92)	1.12 (0.60–2.09)	0.98 (0.50–1.94)
			
Health insurance			
Covered versus Uncovered	2.09 (0.71–6.18)	1.75 (0.77–4.00)	1.95 (0.92–4.17)
			
Agreement with cancer fatalistic beliefs			
Belief 1 Agree versus Disagree	3.34 (1.17–9.51)	1.02 (0.62–1.68)	1.29 (0.71–2.34)
Belief 2 Agree versus Disagree	0.67 (0.22–2.05)	1.13 (0.58–2.18)	0.79 (0.38–1.62)
Belief 3 Agree versus Disagree	1.19 (0.40–3.52)	1.14 (0.67–1.93)	0.77 (0.40–1.47)
Belief 4 Agree versus Disagree	2.56 (0.95–6.90)	0.87 (0.52–1.43)	1.17 (0.64–2.15)
Belief 5 Agree versus Disagree	0.53 (0.20–1.39)	1.85 (1.12–3.05)	NA
			
Belief 5 Agree versus Disagree (in women)	NA	NA	2.65 (1.09–6.44)
Belief 5 Agree versus Disagree (in men)	NA	NA	0.51 (0.21–1.22)

Note

Belief 1: It seems like everything causes cancer.

Belief 2: There is not much you can do to lower your chances of getting cancer.

Belief 3: There are so many different recommendations about preventing cancer, it is hard to know which ones to follow.

Belief 4: When I think about cancer, I automatically think about death.

Belief 5: Cancer is most often caused by a person's behavior or lifestyle.

N/A, not applicable.

A total of 468 respondents were eligible for colorectal cancer screening according to the ACS cancer screening guideline. Among these screening‐eligible respondents, 38.0% ever had a blood stool test and 78.4% ever had a sigmoidoscopy or colonoscopy. Controlling for the other variables, agreement with “Cancer is most often caused by a person's behavior or lifestyle” was associated with a higher likelihood of having had a blood stool test (OR = 1.85; 95% CI: 1.12–3.05), as well as a higher likelihood of having had a sigmoidoscopy or colonoscopy, but among women only (OR = 2.65; 95% CI: 1.09–6.44). Regarding the sociodemographic variables, older age was associated with a higher likelihood of having had a blood stool test (OR = 1.08; 95% CI: 1.04–1.11) or a sigmoidoscopy or colonoscopy (OR = 1.08; 95% CI: 1.03–1.12). Having more than high school education was associated with a higher likelihood of having had a sigmoidoscopy or colonoscopy (OR = 1.90; 95% CI: 1.06–3.41). Respondents with a household income of $75,000 or more were more likely to have had a sigmoidoscopy or colonoscopy (OR = 2.28; 95% CI: 1.05–4.93) compared to those with a household income lower than $35,000.

## 4 DISCUSSION

### 4.1 Principal findings

Based on the RDD telephone survey data collected in the residents of north‐central Florida, we found multiple sociodemographic disparities in cancer fatalistic beliefs. Furthermore, controlling for the respondents’ sociodemographic characteristics, we found that the agreement with “It seems like everything causes cancer” was associated with a higher likelihood of having had breast cancer screening using mammogram, while the agreement with “Cancer is most often caused by a person's behavior or lifestyle” was associated with a higher likelihood of having had colorectal cancer screening using the blood stool test. In addition, we observed a sex by cancer fatalistic belief interaction when predicting the use of sigmoidoscopy or colonoscopy. The agreement with “Cancer is most often caused by a person's behavior or lifestyle” was associated with a higher likelihood of having had a sigmoidoscopy or colonoscopy among women, but the association was non‐significant among men. We did not observe any rural–non‐rural disparity in the association between cancer fatalistic beliefs and cancer screening utilization.

### 4.2 Sociodemographic disparities in cancer fatalistic beliefs

Overall, our results and the literature show great sociodemographic disparities in cancer fatalistic beliefs, but there seem to be no consistent trends in these disparities. Using sex disparity as an example, Kobayashi et al examined the 2017 national HINTS data and reported that women were more likely than men to agree with “It seems like everything causes cancer,” but not the other belief statements, in both univariate analysis and multivariable analysis controlling for other sociodemographic factors.^17^ However, a recent survey study of Texas residents did not find the same sex differences in a similar multivariable analysis but reported that men were more likely than women to agree with “Cancer is most often caused by a person's behavior or lifestyle.”^18^ In another recent study of the residents of Appalachian counties, Vanderpool et al. found that men scored lower than women on a cancer belief agreement index,^19^ indicating that men were, in general, less likely than women to agree with cancer fatalistic beliefs. The sex differences in fatalistic beliefs observed in our study are consistent with those reported in the Texas study but less so with results from the other studies. A plausible explanation is that Florida and Texas are both southern states and share some similarities in social, cultural, and religious characteristics. Nonetheless, current evidence does not seem to support any consistent trends in the sociodemographic disparities in cancer fatalistic beliefs, which appear to vary greatly by geographic region.

### 4.3 Cancer fatalistic beliefs and cancer screening utilization

Our results show that the agreement with the belief “Cancer is most often caused by a person's behavior or lifestyle” is associated with a higher likelihood of getting colorectal cancer screening (among women only for sigmoidoscopy or colonoscopy). Previous research has reported a positive association between holding this belief about how one's behavior can enhance cancer risk and having cancer knowledge, self‐efficacy, and perceived control,^12^, ^28^, ^29^ which may have played an important role in increasing colorectal cancer screening in our study population. However, most prior studies did not find a significant relationship between cancer fatalism and colorectal cancer screening in multivariable analyses. Idowu et al. showed that having fatalistic beliefs was not associated with being up‐to‐date with colorectal cancer screening using the 2007 HINTS data.^30^ Fernández et al. observed a similar non‐significant relationship among Latinos in South Texas.^31^ In the one study that reported a positive relationship between cancer fatalism and colorectal cancer screening, Crosby and Collins discovered that, among the residents of rural Kentucky, holding the belief “There is nothing I can do to reduce my risk of developing colorectal cancer” was associated with a lower likelihood of getting an endoscopy,^32^ whereas a fatalism composite score was not associated with returning a fecal immunochemical test kit.^33^

Our results also show that agreement with the belief “It seems like everything causes cancer” is associated with a higher likelihood of getting breast cancer screening using a mammogram. This finding seems counterintuitive as the literature suggests that individuals who hold such a pessimistic belief are less likely to engage in cancer prevention behaviors.^12^, ^13^ Future research that applies health behavior theories in more sophisticated study designs (e.g., structural equation modeling) is needed to examine the potentially complex relationships among the fatalism beliefs in predicting cancer screening utilization. For example, agreement with this statement could represent the recognition of one's cancer risk and therefore be logically related to obtaining cancer screening, if one has access to it. Further, future research is needed to explore additional social and cultural factors (e.g., religion) that could impact both fatalistic beliefs and breast cancer screening. Similar to the case of colorectal cancer screening, most prior studies did not find a significant relationship between cancer fatalism and breast cancer screening in multivariable analyses.^34^, ^35^, ^36^

### 4.4 Strengths and limitations

Our study has several strengths. This is the first study to examine the relationships among sociodemographics, cancer fatalistic beliefs, and cancer screening utilization in the north‐central Florida population. The unique relationships observed among these variables demonstrate that more research is needed to better understand the social and cultural factors associated with cancer screening utilization in this region. Our study is also of several limitations. First, focusing on a specific geographic location, our study results are not generalizable to the other regions in the US. However, given the unique sociodemographic characteristics of north‐central Florida, our study provides important information for future studies that aim to examine the local context in cancer fatalistic beliefs and cancer prevention behaviors. Second, similar to all the other survey‐based studies, our data analysis was based on self‐reported data that are known to be subject to recall bias and response bias. Third, due to the limited length of telephone surveys, we were unable to include and test all potential risk factors that are relevant to cancer fatalistic beliefs and cancer screening behavior. Some relevant risk factors may be omitted from our analysis. This limitation of our study represents opportunities for future research. Fourth, some model estimates (e.g., ORs for Hispanic origin and certain interaction contrasts involving rural residency) might be underpowered due to small sample sizes. Last, the cross‐sectional nature of the study limits our ability to make any causal inference.

### 4.5 CONCLUSION

Some, but not all, cancer fatalistic beliefs are associated with getting breast and colorectal cancer screening in north‐central Florida, a region characterized by high poverty and high cancer burden. Our study highlights the need for more future research to better understand the social and cultural factors associated with cancer screening utilization in Florida.

## ETHICAL APPROVAL STATEMENT

This study was approved by the University of Florida Institutional Review Board.

## CONFLICT OF INTEREST

The authors declare no conflicts of interest.

## Data Availability

Research data are not shared.
